# Supplementary material for: The effect of human mobility restrictions on the COVID-19 transmission network in China
Source: PLoS One. 2021 Jul 19;16(7):e0254403. doi: 10.1371/journal.pone.0254403 (PMC8289089; doi:10.1371/journal.pone.0254403)
Supplement: Attachment — Submitted filename: R2R.pdf [file pone.0254403.s003.pdf]

Econometrics and Business Statistics
Monash Business School
Monash University
Caulfield East, Vic 3145, Australia

Dear Prof. Bing Xue,

"The Effect of Human Mobility Restrictions on the COVID-19 Transmission Network in China" (Manuscript ID: PONE-D-21-04530)

We would like to begin by thanking your and the reviewers for the extremely useful comments on our manuscript. We are grateful for the opportunity to submit a revised version.

We have addressed all of the points raised by the reviewers, and feel the changes greatly improves the clarity of the manuscript. We have highlighted the changes in one of our manuscripts. Also, we have detailed how we have addressed each comment in our responses separately for the Editor and the two referees.

Thank you again for your help with this submission, and we hope that this revised version is suitable for publication in PLoS One.

Yours sincerely,

Tatsushi Oka (on behalf of all co-authors)
June 13, 2021

Response to the Editor

Journal Requirements

Please review your reference list to ensure that it is complete and correct. If you have cited papers that have been retracted, please include the rationale for doing so in the manuscript text, or remove these references and replace them with relevant current references. Any changes to the reference list should be mentioned in the rebuttal letter that accompanies your revised manuscript. If you need to cite a retracted article, indicate the article's retracted status in the References list and also include a citation and full reference for the retraction notice.

Response: Thank you for highlighting the reference list. We have checked our reference list and it is now complete and correct. In this revision, we additionally cite two papers, which we found after our previous submission. Both of the papers use the SIR-type model for different policy analysis. More precisely, the footnote 6 of the revised paper has the following sentence, "See [32, 33] among others for analysis of other type policies.", and our reference includes the following items:

[32] Acemoglu D, Chernozhukov V, Werning I, Whinston MD. Optimal targeted lockdowns in a multi-group SIR model. vol. 27102. National Bureau of Economic Research; 2020.

[33] Callaway B, Li T. Policy Evaluation during a Pandemic. arXiv preprint arXiv:210506927. 2021;

Response to Reviewer 2's Comments

After revision, the quality of this paper has been improved. The author deals with the comments within his ability. I recommend that this paper be published in PLoS ONE with minor amendments.

The results of this paper provide useful information about COVID-19 transmission network in China. However, the discussion part and conclusion part are still not well written.

Response: We are grateful to the referee for detailed comments again. In the revision, we have addressed the points you raised regarding the discussion and conclusion part. We hope that the revised version will meet the standard. The following is a detailed response to your comments and suggestions and how we revised the paper.

First, the part of the discussion is incomplete. Please refer to my first round of comments.

Response: In this revision, we strictly follow your comments in the first round:

> The section of Discussion in a paper is generally involved with 3 or 4 parts: (1) main points, which response to the questions put in introduction; (2) comments on related studies or problems; (3) shortcomings or deficiency in study method or process; (4) conclusions, which can be separated to make the final section.

The discussion part now consists of three paragraphs, each of which corresponds to the item (1)-(3) in order, as follows.

> (1) main points, which summarize the purpose of our study and our main findings.
>
> In this paper, we analyze the propagation of COVID-19 among 33 regions in China. We develop a spatial model that extends the SIR-type model to estimate the effect of the policy interventions on the disease spread across 33 regions. Our estimation results suggest that secondary epicenters such as Beijing, Guangdong, and Shanghai, developed at a very early stage of the outbreak. Our analysis also shows that mobility restrictions across regions indeed prevented the further spread of the disease. Community transmission was observed to be the primary source of infection, and it declines substantially following local policy interventions.
>
> (2) comments on related studies, in particularly the limitations of existing studies compared to our proposed method.
>
> The epidemiology literature traditionally focuses on deterministic SIR-type models, while our paper extends the standard models to allow for a stochastic mechanism. Also, since the COVID-19 outbreak, a few studies have used the Baidu database to document the disease transmission, but they only focus on the transmission from the primary

epicenter, Wuhan, to the rest of China. Our spatial transmission network suggests that other epicenters can quickly become established.

(3) shortcomings of our analysis. The conclusions are covered in a separate part.

Due to the lack of data availability, our analysis in this paper does not separately evaluate the effects of different policies among regions. If detailed data is available, however, our model does allow such analysis.

Second, the value of this study cannot be seen from the section of conclusions. The conclusion part does not give useful information about COVID-19 transmission network in China.

Response: Thank you for the comment. We now explain in the Section of Conclusions that our transmission network allows us to differentiate the effect of local policies and cross-region mobility restrictions, which leads to the conclusion that both local lockdown policies and cross-region mobility restrictions are important for curbing the transmission. We also highlight the value of our transmission network in the abstract and the conclusion part in the introduction.
