# Supplementary material for: The effect of human mobility restrictions on the COVID-19 transmission network in China
Source: PLoS One. 2021 Jul 19;16(7):e0254403. doi: 10.1371/journal.pone.0254403 (PMC8289089; doi:10.1371/journal.pone.0254403)
Supplement: Attachment — Submitted filename: R2RPONE-D-21-04530.docx [file pone.0254403.s002.docx]

Response to the Editor

Journal Requirements

Please ensure that your manuscript meets PLOS ONE’s style requirements, including those

for file naming. The PLOS ONE style templates can be found at

Response: Thank you for highlighting PLOS ONE’s style requirements. We have now

amended the manuscript to align with these guidelines.

In the Methods section please provide a citation for the Baidu mobility data, and please

provide further clarification whether this is a publicly accessible database. Furthermore,

please specify the date range over which data was extracted.

Response: Thank you for pointing out that the data source is not cited in Section Model,

although it is cited in Section Introduction and Data. We now cite the data source in

Subsection External Transmission as well. We have also added a sentence in Subsection

Data to indicate that the data is publicly accessible.

Please provide an amended statement that declares *all* the funding or sources of support

(whether external or internal to your organization) received during this study, as detailed

online in our guide for authors at http://journals.plos.org/plosone/s/submit-now. Please also

include the statement “There was no additional external funding received for this study.” in

your updated Funding Statement.

Please include your amended Funding Statement within your cover letter. We will change

the online submission form on your behalf.

Response: We include all funding source, which is the same as the one for our fist submission.

2

Response to Reviewer 1’s Comments

We appreciate your positive and considerate comments, which greatly improved our paper. Please

find the response to your comments below.

1. The four special admisnistrative areas in China is offically called Municipalities, which include

Beijing, Shanghai, Tianjin, and Chongqing.

Response: Thank you for pointing out this point. We revised footnote 1 of page 2 so that

those four places are explained as municipalities. For simplicity, we call cross-sectional units

as “regions” throughout the paper.

2. In Figure 3, the patern in Hong Kong is so different from other regions. Please explain

about the special reason, possibly the difference on the policy. It is a good point to make

comparisons.

Response: Thank you for highlighting the differences in estimation results between Hong

Kong and the other regions. There are several possible reasons why infections patterns in

Hong Kong are different from the other regions. As you pointed out, Hong Kong has different

policies from the other regions for quarantine, school suspension, social distancing rules and so

on. Also, Hong Kong is special geographically, being a premier trade and travel hub in Asia.

These characteristics could potentially explain the differences. We have added Subsection

“China’s practices to contain COVID-19 transmission” to discuss how the polices adopted

in Hong Kong differred from other regions in China, and explined that the different policies

likely affected the internal transmission rate in Subsection “Transmission Rates: External

versus Internal”.

3. Are there differences on policies in regions? How about the effectiveness?

Response: We have information about timings of policies implemented in all regions of China,

but we do not have reliable information on detailed differences of the polices across all regions

in China, nor their effectiveness. The different timing of policy implementation is reflected in

the variable Xj;t􀀀h, in equation (4), page 6. To take into account the possible differences and

effectiveness of the policies, our model allows for heterogeneous policy effects _j for region j

in equation (4), page 6. Thus, if local policies have different policies and effectiveness, our

estimation results reflect them.

4. It is possible to itemize the restrictions like highway, railway, and airlines?

3

Response: We added some discussion on restrictions that local governments imposed in

Subsection “China’s practices to contain COVID-19 transmission”. However, we do not have

reliable information covering the whole of China, while the information is anecdotal evidence.

We provide relevant information in the subsection, while our model cannot incorporate the

information.

4

Response to Reviewer 2’s Comments

We are grateful to the referee for detailed and thoughtful comments. They were helpful in improving

the paper. In the revision, we have addressed all comments as best as we could. The points

you put forward were very useful for us to revise our previous work and improve the quality of this

paper. The following is a detailed response to your comments and suggestions and how we revised

the paper.

1. The structure of Abstract is not complete: The abstract was not well organized in this paper.

A good abstract comprises the following four parts: background, methods, results or findings,

conclusions or significance. The components of this paper’s abstract is not complete.

Response: Following your suggestion, we have changed the format of the abstract so it now

explicitly itemizes background, methods, result, and conclusions.

2. Figures: In order to make the content of a plot clear, the text labels should be added to both

the x- and the y- axes (e.g., figure 1-3).

Response: We added text labels on horizontal and vertical axes of Figures 1-3.

3. Models: The theoretical basis of the model presented in the paper is not clear. As we

know, mathematical models fall into two types: mechanism type and parameter type. Correspondingly,

the modeling method can be divided into two categories: analytical method

and experimental method. What method does the author used in this article? What model

to build? The author’s modeling idea and process are not clear enough.

Response: As explained in Section “Model”, the basis of our model is the susceptible-infectiverecovered

(SIR) model. The SIR model is one of well-established models in the Epidemiology

literature and has been used to describes dynamic transition across the susceptible, infective

or recovered compartments. There are a variety of extensions of the SIR model, including

Fibich (2016), who considers the infection rate changes according to internal and external

factors. This extension is inspired by the Bass model (Bass, 1969), which is an influential

model for describing the diffusion of new products. Given the development of the SIR

model and its extensions, our model can be viewed as a model describing the mechanism of

propagation of infectious diseases.

We are not familiar with the precise definition of mechanism type and paramter type models

and what the difference is between the two, which may be partly due to differences between

our fields. However, we attempt to provide a comprehensive response to your question. As

5

we explained above, the model we consider describes the propagation mechanism of infectious

diseases, while it has parameters that can be interpreted in the context of a SIR type model.

In terms of how the SIR model accurately describes the transmission mechanism of infectious

diseases, unlike physics or engineering fields, the field of Statistics or Economics builds models

to approximate the reality which is far more complex in truth than any model. This leads

to mis-specification in the models which may be the case for the SIR model in that it does

not describe disease transmission at individual level but instead describes phase transition

at the population level. As shown in the existing literature, however, even when a model is

mis-specified, it can be a useful tool to describe or understand the reality, and the model

parameters can be considered as pseudo parameters (Box, 1976; White, 1982). A large body

of literature in Epidemiology and Statistics have shown that the SIR model and its extensions

are useful to understand and predict infectious diseases. In fact, the usefulness of the SIR

has been demonstrated for the current pandemic and our research aims to contribute to the

literature along this line.

In terms of the questions of whether the method is analytical or experimental, we think that

the estimation method of SIR model is not regarded as an experimental approach. What the

SIR model attempts to do is to explain the observed phenomena in the real world, rather

than the data generated by randomized control trials or other experimental settings. It is

always better to have experimental data to estimate models, despite the fact that running

experiments in the real world is costly and sometimes also ethically problematic, in particular

for infectious disease. Thus the literature in Epidemiology, Statistics and Economics have

made progress in developing models and estimation methods to meet available data and

information.

Overall, we appreciate your stimulating question regarding models, which has encouraged

us to consider how we can see reality through the lens of models. Because models play

different roles depending on the available information, observed data as well as uncertainty

surrounding models, setting models and their interpretation can differ across fields. We hope

that you understand that our modeling approach may be different from the approach in your

field, possibly due to the limitation of information and data. We think that coexistence

of different approaches for modeling should be beneficial rather than harmful in that they

enrich our understanding of the real world from different angles.

4. Results and conclusions: Conclusions are different from results. Generally speaking, results

come from or are directly based on data analysis. In contrast, conclusions come from discussion

and represent the climax of discussion. If the result is regarded as the heart of an

6

academic paper, the discussion can be treated as the nerve center of the paper. Discussions

form a bridge between results and conclusions. In this paper, the results are confused with

conclusions.

Response: Following your suggestion, we now clearly separate results and conclusions in

the abstract. In the last section, we added discussions of the results, which focuses on how

transmission evolved in response to government policies in our data, and then present the conclusions,

which focuses on the more general policy implications for infectious disease. Thank

you for the suggestion, which enables our paper to distinguish our results and conclusion

clearly.

5. Lack of discussion: The section of Results represents the heart of a paper, and the section

of Discussion is the paper’s nerve center. [See: Robert A. Day, Barbara Gastel. How to

Write and Publish a Scientific Paper (Sixth Edition). Cambridge University Press, 2003]

The analytical process of discussion is not clear. The section of Discussion in a paper is

generally involved with 3 or 4 parts: (1) main points, which response to the questions put in

introduction; (2) comments on related studies or problems; (3) shortcomings or deficiency in

study method or process; (4) conclusions, which can be separated to make the final section.

[See: Bjorn Gustavii (2002). How to Write and Illustrate a Scientific Paper. Cambridge:

Cambridge University Press]

Response: Thank you for your detailed advice and the relevant references. We have revised

the paper so that the discussion part in the final section covers the items you raised.

6. Conclusions are not well extracted: The conclusions should be made clearer in expressions

and meanings. The important conclusions should be given three times in a paper: once in the

Abstract, again in the Introduction, and again (in more detail probably) in the Discussion

(if it contains a Conclusion paragraph) or Conclusions (if this part is separated as the final

section). [See: Robert A. Day, Barbara Gastel. How to Write and Publish a Scientific Paper

(Sixth Edition). Cambridge University Press, 2003]

The problem with this article is that there is no significant difference between the section

of conclusion part and the section of abstract. As indicated above, a complete abstract

comprises four components: background and aim, method, results, and conclusions.

Response: The revised version of our paper now has a section dedicated to our conclusions.

We also state the most important conclusions in the abstract and in the last paragraph

Section “Introduction”. Thank you for the suggestion, which makes our conclusions clearer.

7
